# Quality preservation of jujube fruit during cold storage: effect of maturity stage and modified atmosphere packaging

**DOI:** 10.1186/s12870-025-07492-9

**Published:** 2025-10-21

**Authors:** Sefa Gun, Burhan Ozturk, Erdal Aglar, Orhan Karakaya

**Affiliations:** 1 Department of Horticulture, Faculty of Agriculture, Ordu University, Ordu, Türkiye; 2 Department of Landscape Architecture, Faculty of Arts, Design and Architecture, Munzur University, Tunceli, Türkiye; 3 Department of Horticulture, Faculty of Agriculture, Sakarya University of Applied Sciences, Sakarya, Türkiye

**Keywords:** Antioxidant activity, Total flavonoids, Total phenolics, Respiration rate, Vitamin C

## Abstract

**Bacground:**

This study, which examines the effect between the maturity stage and modified atmosphere packaging (MAP) in order to prevent post-harvest quality losses and extend the storage life of jujube (*Ziziphus jujuba* Mill.) fruit, offers an important contribution to supply chain management. The fruits harvested at different maturity stages were stored at 0 ± 0.5 °C and 90 ± 5 RH in MAP and control conditions for 42 d and changes in quality, physiological and biochemical parameters were evaluated.

**Results:**

The lowest value was recorded in the early maturity (M-1) + MAP group for weight loss. Respiration rate decreased with advancing maturity, MAP treatments slowed down this process. Oxygen consumption and CO₂ production changed according to maturity level, MAP applications balanced gas exchange. MAP maintained brightness and color intensity of the fruit better. It also slowed down the firmness loss. The highest soluble solid content (SSC) was determined in the late maturity (M-3) + MAP group. Titratable acidity (TA) showed lower increase in MAP treatments. MAP significantly reduced the losses of vitamin C. Total phenolic, total flavonoids and antioxidant activity decreased during storage, while MAP treatments limited this decrease.

**Conclusions:**

The findings revealed that packaging jujube fruit with MAP, especially in early and mid-maturity stages, better preserves its quality parameters. Accordingly, MAP applications appropriate to the ripeness stage are recommended as an effective strategy to extend the fresh consumption period of jujube fruit and preserve its bioactive compounds.

## Background

Jujube (*Ziziphus jujuba* Mill.) is a valuable fruit used in both functional food and traditional medicine due to its richness in vitamin C, polyphenols, flavonoidss and various antioxidant compounds [1]. Jujube, which is widely consumed both fresh and dried due to its sweet flavor and nutritional content, attracts attention with its immune system supporting, anti-inflammatory and antiging effects [2]. In recent years, its consumption, especially in fresh form, has increased, which has further increased the commercial importance of the fruit. However, jujube fruit is quite prone to quality losses such as rapid water loss, tissue softening, color change, ethanol fermentation and decay in the post-harvest period [3, 4]. Its high respiration rate and climacteric nature further accelerate these quality deteriorations, limiting the marketability of varieties suitable for fresh consumption [5]. Although cold storage is generally used to slow down this process, it is not sufficient on its own and can lead to secondary problems such as low temperature damage [6]. Therefore, more effective and integrated preservation strategies are needed to prevent quality loss in the post-harvest period.

One of the main factors affecting the storage performance of jujube fruit is harvest maturity. Maturity stages classified based on rind color (white, semi-ripe, fully ripe) are directly determinant on the tissue durability, sugarcid balance, phenolic compound content and consumer acceptance of the fruit [7]. Although fruits harvested at early maturity are more advantageous in terms of shelf life, they are insufficient in terms of taste and aroma; those harvested at full maturity are delicious but more prone to spoilage [8]. This makes it difficult for producers to determine the ideal harvest time and affects the market value of the product. As ripening progresses, the significant structural changes occur in the fruit cell wall, and the activity of hydrolytic enzymes such as PG, PME, β-Gal, cellulase and XET increases, accelerating fruit softening [9]. At the same time, the phenolic compounds and antioxidant activities are also observed to vary according to different ripening stages [10]. Therefore, the detailed examination of the biochemical and physiological changes that occur in the fruit depending on the maturity stage is of critical importance in determining the level of success of interventions to preserve quality [11].

One of the most effective technologies to slow down postharvest quality losses is MAP application. MAP slows down fruit respiration by changing the gas composition in the package, limits ethylene production and delays aging processes by reducing oxidative stress [12]. It has been determined that this technology prevents water loss, firmness loss, color change and decay in sensitive fruits such as jujube; and also reduces textural deterioration by suppressing the activity of cell wall enzymes such as PPO, PG and PME [13]. It has also been reported that MAP contributes to the preservation of phenolic compounds and ascorbic acid and helps the fruit cope with metabolic stress by regulating the redox balance [5]. However, the existing studies have generally focused on a single maturity stage, and the effectiveness of MAP at different maturity levels has not been systematically evaluated [14]. However, in climacteric species such as jujube, the rapid changes in respiration and metabolic processes with maturity directly affect the effect of MAP. In this context, the need for comprehensive studies evaluating the effect of maturity stage and MAP together is quite clear. The aim of this study was to determine the effect of MAP applied to jujube fruit harvested at different maturity stages on quality properties and bioactive compounds during cold storage. In this context, it is aimed to extend the shelf life and reduce nutritional and quality losses by optimizing the appropriate maturity stage and MAP strategy together. It is hypothesized that MAP treatment will significantly reduce postharvest quality losses in jujube fruit, and that the effectiveness of MAP in preserving quality and bioactive compounds will vary depending on the harvest maturity stage.

## Materials and methods

### Plant material

This study was carried out with fruits obtained from five-year-old *Ziziphus jujuba* Mill. cv. ‘Lang’ variety jujube trees located in Yeşilvadi Farm in Harmanağılı Village of Suluova district of Amasya province (40°44′25.35" N, 35°45′26.12" E, 415 m altitude). The experimental orchard was established with seedlings propagated from suckers and the trees were planted in the south-north direction with 5.0 × 2.0 m spacing and trained according to the central leader system. The trees were reinforced with a wire support system and irrigated with a double-line drip irrigation system. All cultural practice (pruning, fertilization, irrigation etc.) were performed regularly.

### Methods

In the study, jujube fruits were harvested manually from 30 trees at three different maturity stages according to the color change of the fruit peel surface [Maturity 1 (M-1, 0–10% reddish of fruit surface), Maturity 2 (M-2, 11–50% reddish of fruit surface) and Maturity 3 (M-3, > 51% reddish of fruit surface)]. To strengthen this classification, initial SSC and fruit firmness values at harvest were also recorded and used as supporting physiological indicators for the maturity grouping. Approximately 30 kg of fruit was harvested randomly from 30 trees and mixed within each maturity group. After harvest, the fruits were transported to Ordu University, Faculty of Agriculture, Department of Horticulture laboratory by refrigerated vehicle (10˚C and 90% RH) within 3.5 h. Fruits in each maturity group were evaluated under two different conditions: control and MAP treatment. For each maturtiy stage (M-1, M-2, M3), 500 g fruit were placed 36, in total 108 polyethylen packages containers (1000 mL, Vempi, Türkiye), without clamshell, and with 4 holes of 1 cm diameter. Half of the containers (54) were placed in Xtend® (815-CH97/a, StePac, Türkiye) MAP packaging, and the other half were left as the control group. Xtend® bags were used in the MAP groups, while the control group was stored in the same polyethylene containers with holes and without gas modification that is, exposed to normal oxygen and carbon dioxide levels at ambient conditions. This allowed for a meaningful comparison between the control and MAP treatments. Three treatments (M-1 + MAP, M-2 + MAP and M-3 + MAP) were applied to the fruits. Oxygen (O_2_) and carbon dioxide (CO_2_) ratios during storage in MAP packages ranged from 20.50%−19.50% O_2_ and 0.05%−0.25% CO_2_ for M-1 + MAP; 20.20–19.70% O_2_ and 0.05%−0.25% CO_2_ for M-2 + MAP; 19.80%−19.50% O_2_ and 0.10%−0.20% CO_2_ for M-3 + MAP. The fruits were pre-cooled at + 4 ± 0.5 °C with 90 ± 5% RH for 24 h. The MAPs were then closed with plastic clips, and all the fruit was stored in cold storage at 0 ± 0.5 °C and 90 ± 5% RH for 42 days. At harvest and during the storage period (7, 14, 21, 28, 35 and 42 days), the following quality parameters were evaluated in the fruits of each treatment at weekly intervals.

### Weight loss

The weight of the fruits was measured using a high-precision digital balance (accuracy ± 0.01 g; Radwag PS 4500/C/1, Poland). The mass reduction during the cold storage period was calculated by taking into account the difference in weight before and after storage in each replicate and expressed as a percentage (%).

### Respiration rate

The amount of CO_2_ released to the external environment by 5 fruits, when kept in a 2 L closed gas-tight glass container at 23 ± 1.0 °C and 90% relative humidity for 1.0 h, was measured with a digital carbon dioxide sensor (Vernier, Oregon, USA) and calculated as mL CO_2_ kg^−1^ h^−1^ based on the weight and volume of the fruits placed in the glass container. In addition, O_2_ and CO_2_ concentrations in MAP-applied fruits will be measured weekly with an analyzer (Abiss Legend, France) and expressed as percentage [12].

### Fruit color

Fruit peel color was determined in terms of CIE L*, a* and b*. The values ​​of color characteristics were determined in 10 selected fruits by taking measurements from the two opposite poles of the equatorial part of the fruit in each analysis period of cold storage measurements using a colorimeter (Minolta, model CR-400, Tokyo, Japan). According to the prepared scale, the a* value is expressed as redness-greenness, and the b* value is expressed as yellownesslueness. The chroma value = (a*^2^ + b*^2^)^1/2^, and the hue angle value is determined by the formula hº = tan^−1^ × b*/a*.

### Fruit flesh firmness

Fruit flesh firmness was determined with an Agrosta®100Field digital firmness meter from 2 different sides of the equatorial part of 10 fruits in each replicate and the values ​​were expressed in Newton (N).

### Soluble solids content, titratable acidity and vitamin C

In each replication, one slice was taken from 10 fruits and the homogenate obtained after being squeezed by an electric juicer was passed through a cheesecloth and the juice was obtained. For SSC, the sufficient amount of the juice sample was taken and readings were made with a digital refractometer (PAL-1, McCormick Fruit Tech. Yakima, USA) and the values were expressed as percentage. For TA, 10 mL of the fruit juice sample was diluted with 10 mL of distilled water water and then titrated with 0.1 mol L-1 (N) sodium hydroxide (NaOH) until the pH value reached 8.1, and the amount of NaOH consumed in the titration was expressed in terms of malic acid (g malic acid 100 mL^−1^). Reflectoquant plus 10 brand device (Merck RQflex plus 10, Germany) was used for the determination of vitamin C. After the fruit juice obtained for the SSC measurement was diluted tenfold with oxalic acid (5 g fruit juice sample, 50 ml oxalic acid), the ascorbic acid test kit was immersed in the diluted solution for 2 s, kept outside for 8 s to oxidize, and then placed into the test adapter of the Reflectoquant device for 5 s. The value read on the device was recorded and expressed as mg 100 g^−1^ [12].

### Sample preperation of bioactive compounds

In each analysis period, approximately 10 fruits from each replicate of each treatment were washed with distilled water and dried at room temperature. Then, the seeds of the fruits were removed and sliced with a stainless knife and homogenized with a food blender. The homogenized fruit samples were placed in falcon tubes (approximately 75–100 g) and stored at −80 °C until the bioactive analyses specified below were performed.

### Total phenolics

Total phenolic content was determined according to the method of Singleton and Rossi [15]. 500 µL of the stock solution was taken and 4.1 ml of distilled water, 100 µL of folin reagent and 300 mL of sodium carbonate were added. After incubation for two hours, the samples were read at 760 nm in a spectrophotometer (Shimadzu, Kyoto, Japan). The results were expressed as mg GAE 100 g⁻^1^ fresh weight.

### Total flavonoids

Total flavonoids were determined as stated in the study of Ozturk and Ozer [16]. 1000 μL of prepared extract was taken and completed to 4300 μL with methanol. Then, 100 μL of 10% aluminum nitrate [Al(NO_3_)_3_] and 0.1 M ammonium acetate (NH_4_CH_3_CO_2_) were added to the solution, respectively. The samples were read at 510 nm in a spectrophotometer. Total flavonoids content was expressed as quercetin equivalent (QE), mg QE 100 g^−1^ fw fresh weight.

### FRAP assay

The buffer solution for the FRAP analysis was prepared by mixing 0.1 mol/L acetate buffer (pH 3.6), 10 mmol/L TPTZ (2,4,6-tripyridyl-s-triazine, dissolved in 40 mmol/L HCl), and 20 mmol/L FeCl₃·6H₂O at a 10:1:1 ratio. For the measurement, 2.98 mL of FRAP reagent was mixed with 20 µL of fruit extract. The mixture was incubated at 37 °C for 10 min before being read on a spectrophotometer at a wavelength of 593 nm. The standard curve was obtained using Trolox solutions prepared in the range of 0–1000 µmol/L. Results were calculated according to the standard curve and expressed as mmol Trolox equivalents (TE) per 100 g fresh weight [17].

### DPPH assay

DPPH radical scavenging activity was determined using a modification of the Blois [18] method. A 0.1 mmol/L DPPH solution was prepared in 96% ethanol. For analysis, 100 µL of fruit extract was mixed with 2.9 mL of ethanol and 1.0 mL of DPPH solution. The mixtures were incubated in the dark at room temperature for 30 min before absorbance was measured at 517 nm using a spectrophotometer. Trolox was used as the standard substance, and calculations were made according to a standard curve prepared in the range of 0–1000 µmol/L. Results are expressed in mmol TE per 100 g fresh weight.

### Statistical analysis

The statistical design used in our study was a factorial design based on a randomized plot design. Two main factors were considered in the experiment: maturity stage (M-1, M-2, M-3) and treatment (Control, MAP). The combination of these two factors resulted in six treatment groups, each with three replicates. The obtained data were subjected to analysis of variance (ANOVA). Statistical significance of differences between treatments was determined, and significant means were grouped using the Tukey multiple comparison test.The normal distribution control of the data obtained from the research was done with the Kolmogorov-Simirnov test and the homogeneity control of the group variances was done with the Levene test. As a result of the control, the descriptive statistics of the data meeting the conditions were calculated and evaluated with variance analysis. After the data obtained were analyzed with variance analysis (ANOVA), the significance level between the treatments was determined with Tukey's multiple comparison test. Statistical analyses were performed in the SAS package program (SAS 9.1, USA). In statistical analyses and in the interpretation of the results, the significance level was taken into account as α = 5%.

## Results and discussion

### Weight loss

**Fig. 1:**
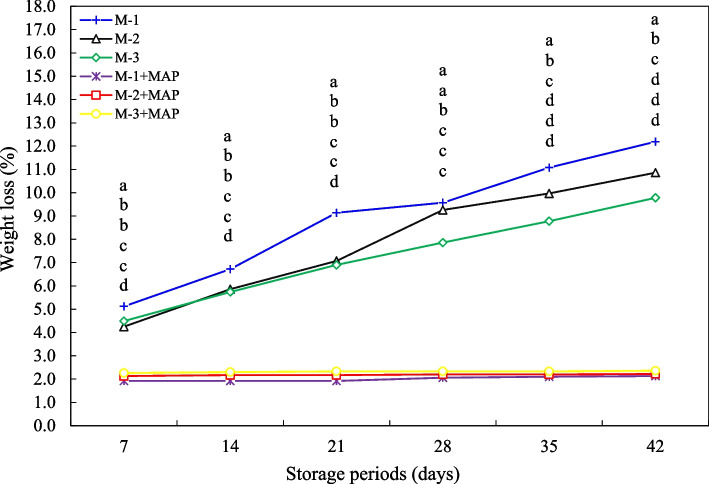
Effect of maturity stage and modified atmosphere packaging on weight loss of jujube fruit during cold storage. Means with the same lowercase letter in the same column are no different from each other (Tukey test, *p* ≤ 0.05)

The effects of harvesting jujube fruit at different maturity stages and MAP on fruit weight loss during cold storage were investigated. The highest weight loss occurred in fruits harvested at least maturity (M-1), and this loss increased throughout the storage period, reaching 12.19% at the end of the 42^nd^ day of cold storage. The weight loss was 10.86% in fruits harvested at mid maturity (M-2), but this value was 9.78% in those harvested at fully maturity (M-3). This suggests that as fruit maturity decreases, the resistance of cell membranes to water loss increases, resulting in less weight loss. The weight loss was significantly reduced in MAP-treated fruits. In particular, in the M-1 + MAP group harvested at least maturity, weight loss at the end of storage was only 2.13% while in fruits harvested at mid and fully maturity (M-2 + MAP, M-3 + MAP), this loss did not exceed 2.36% (*p* ≤ 0.05) (Fig. 1). These findings suggest that MAP preserves quality by inhibiting water loss from the fruit surface and slowing respiration rates, thus extending shelf life. These results support studies by Bernalte et al. [19] that indicate that the resistance of peel tissue to water loss increases with advancing maturity. Similarly, Khaliq et al. [20], Jat et al. [21], and Sen et al. [22] also reported findings that MAP treatment suppresses metabolic activities and reduces weight loss by limiting evaporation in fruit. In our study, the low weight loss values of MAP treatments below 2%, particularly in the first three weeks of storage, are consistent with this literature. In the control groups, the weight loss of nearly 12% was observed after 42 days, well above the 5% limit recommended for commercial marketability (Fig. 1). Therefore, MAP treatment is understood to be a critical quality preservative for fruits with short shelf lives, such as jujubes, especially during cold storage. Researchers such as Ozturk et al. [23] and Khan et al. [24] have also reported that MAP provides superior weight loss control compared to edible coatings in various fruit species. Furthermore, MAP technology is known to similarly prevent water loss and reduce peel wrinkling and decay rates in other fruits such as pomegranates, peaches, cherries, and strawberries [25, 26]. However, high CO₂ and low O₂ levels in some fruits can lead to anaerobic respiration and off-flavor [27], so careful adjustment of gas mixtures is necessary. Microporous packaging systems (MMAP), as suggested by Cui et al. [28] and Qu et al. [29], are among the promising alternatives in this regard.

### Respiration rate

**Fig. 2:**
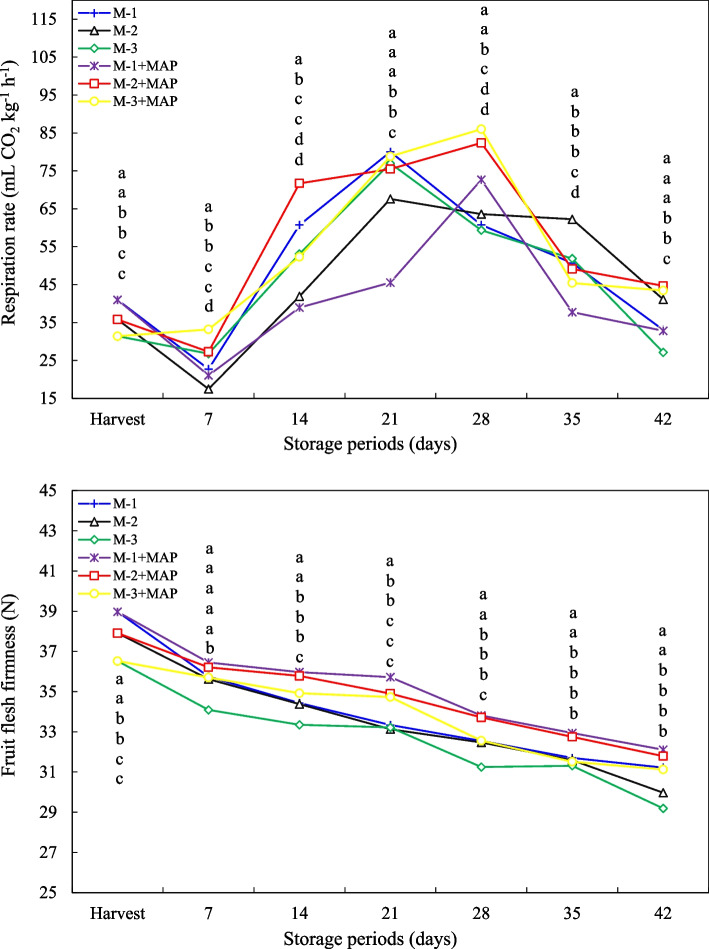
Effect of maturity stage and modified atmosphere packing on respiration rate and fruit flesh firmness of jujube fruit during cold storage. Means with the same lowercase letter in the same column are no different from each other (Tukey test, *p* ≤ 0.05)

At harvest, the highest respiration rate was determined as 40.97 mL CO₂ kg⁻^1^ h⁻^1^ in less maturity fruits (M-1) while mid maturity fruits (M-2) and fully maturity fruits (M-3) showed lower respiration rates of 35.82 and 31.39, respectively. This result indicates that as the fruit ripens, the respiration rate increases due to increased metabolic activity. The fluctuations in respiration rate were observed in all treatments throughout the storage period. In the M-1 group, respiration rate peaked at 80.02 mL CO₂ kg⁻^1^ h⁻^1^ on 21^st^ day and then decreased. Similarly, in the M-3 group, high respiration activity was observed on 21^st^ day with a value of 77.00. In the M-2 group, the peak respiration rate was determined slightly later, at 62.25 on day 35. These findings suggest that metabolic responses and respiration rates at each maturity stage reach their maximum at different times. The effect of MAP treatment varied depending on maturity level. In the M-1 + MAP treatment, the respiration rates were lower than in the control group, particularly during the 7–35 days of storage. It was understood that MAP can delay quality loss by suppressing respiration rates in fully ripe fruits. In contrast, in the M-2 + MAP and M-3 + MAP treatments, respiration rates reached significantly higher levels (71.71–86.01 mL CO₂ kg⁻^1^ h⁻^1^) at some periods (on days 14, 21, and 28) (*p* ≤ 0.05). This suggests that MAP treatment does not always reduce respiration rate; it may stimulate metabolic activity, particularly in fully maturity fruits, depending on gas composition and storage conditions (Fig. 2). These results are consistent with findings in the literature that respiration rate increases due to higher metabolic activity in younger fruits [30]. This finding is consistent with previous studies [31] that MAP treatment delays ripening by suppressing fruit metabolism, thus extending shelf life. However, the data in this study suggest that the effect of MAP is not linear but interacts with fruit maturity and in-package gas composition. MAP applications are reported in the literature to reduce the respiration rate by decreasing the oxygen level and increasing the carbon dioxide level, thus suppressing the Krebs cycle and enzymatic activities [12]. Low oxygen conditions have also been reported to delay fruit softening and deterioration by suppressing ethylene biosynthesis [32]. These mechanisms support the effectiveness of MAP application in regulating respiration, particularly in fully maturity fruit.

### Fruit color

**Fig. 3:**
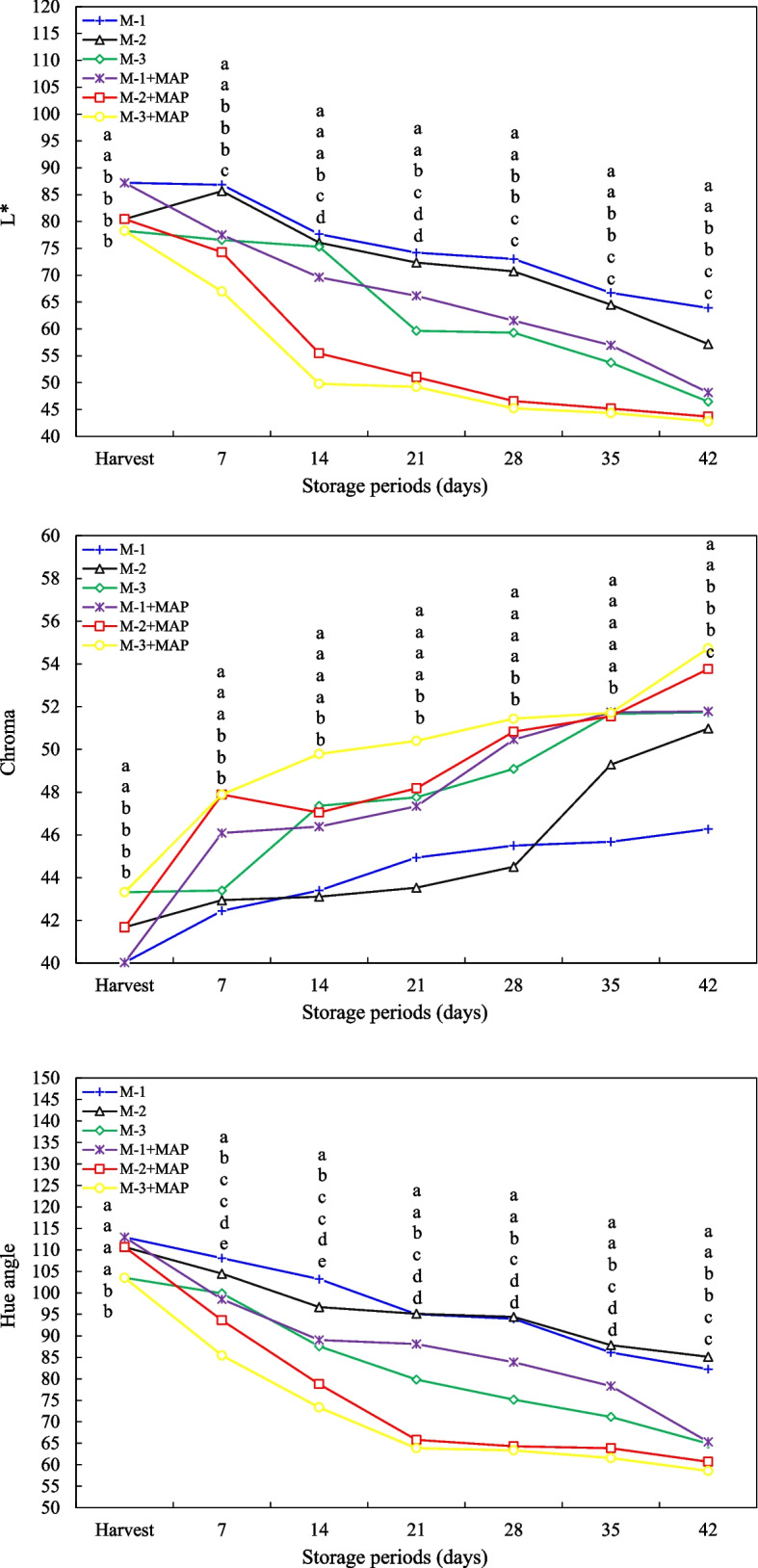
Effect of maturity stage and modified atmosphere packing on L*, chroma and hue angle value of jujube fruit during cold storage. Means with the same lowercase letter in the same column are no different from each other (Tukey test, *p* ≤ 0.05)

In this study, the changes in fruit color values (L*, chroma and hue angle) of jujube (*Ziziphus jujuba* Mill.) fruits harvested at different maturity stages and applied MAP during cold storage. At harvest, the highest L* value (87.21) was found in less maturity (M-1) fruits while these values decreased in less ripe fruits (Fig. 3). As storage progressed, L* values decreased in all treatments, with the lowest openness value (42.79) being reached, particularly in the fully maturity (M-3 + MAP) MAP-treated group. This indicates that the fruit peel darkens and matures during storage (Fig. 3). The chroma values remained lower in less maturity fruits, but increased over time in fully maturity fruits. MAP treatment further increased chroma values, enhancing the fruit's color (Fig. 3). Hue angle data clearly revealed the fruit's transformation from greenish tones to reddish-brown, and MAP treatment accelerated the development of this color tone, particularly after day 28 (p ≤ 0.05) (Fig. 3). These results highlight that fruit color is the critical quality criterion for consumer acceptance and marketability. The decrease in L* during storage may be related to the decrease in brightness and darkening of color with ripening, as reported by Usenik et al. [33]and Khan et al. [34]. The early decrease in L* caused by MAP treatment may indicate that this technology accelerates the fruit ripening process. However, some studies, such as Candir et al. [35], indicate that high CO₂ concentrations in MAP may delay color loss and preserve brightness. The increase in chroma value demonstrates the potential of MAP application to maintain the fruit color. Selcuk and Erkan [36] reported that MAP maintains color intensity by slowing the oxidation of anthocyanins and phenolic compounds; this study supports a similar conclusion. The decrease in hue angle indicates the change in fruit color from green to reddish-brown, and its acceleration by MAP in ripe fruits suggests that pigment conversion is stimulated. Ozturk et al. [37] also noted that red color development is closely related to hue angle. However, Diaz-Mula et al. [38] stated that MAP may suppress color development by delaying maturity, but in this study the such the suppressive effect was not observed; on the contrary, the color change was accelerated by MAP. As a result, MAP treatment accelerated the color change in jujube fruit and positively affected the color saturation and tone development of the fruit peel, thus increasing visual quality and marketability. However, the gas composition and application time must be carefully optimized to avoid the risk of excessive darkening and color loss. As noted by Patino et al. [39] and Ozturk et al. [23], the effect of MAP on color development varies depending on fruit type, atmospheric composition, and storage duration.

### Fruit firmness

The highest fruit firmness value in the harvested fruit was measured as 38.97 N in the M-1 group. As the storage period progressed, the decrease in fruit firmness was observed in all groups and at the end of the 42^nd^ day, the values decreased to 31.22 N in the M-1 group, 29.97 N in M2, and 29.19 N in M-3. The fruit firmness, which was initially lower in fully maturity fruits (M-3), approached that of other groups over time. MAP treatment showed the significant protective effect in maintaining fruit firmness. The fruit firmness loss in MAP-applied fruits was more limited, and at the end of the 42nd day, M-1 + MAP was found to be 32.11 N, M-2 + MAP was 31.79 N, M-3 + MAP was 31.13 N. The statistical analysis showed that MAP treatment significantly slowed down the firmness loss in M-1 and M-2 groups, especially on the 14^th^ and 21^st^ days of cold storage (p ≤ 0.05) (Fig. 2). Fruit flesh firmness is an important indicator of cell wall integrity and turgor pressure and is the critical quality parameter for consumer acceptance and shipping durability [40]. Turgor loss increases with the breakdown of pectin, cellulose, and hemicellulose during the ripening process, leading to a decrease in fruit firmness [41]. The study confirmed that fruit firmness decreases as maturity increases and that this decrease accelerates with storage duration. MAP treatment suppresses ethylene synthesis and cell wall hydrolysis by reducing fruit respiration rate, thus delaying the softening process [42]. Similarly, previous studies by Jat et al. [21] and Avci [43] reported that MAP slowed down the firmness loss in fruits such as jujube and plum. This protective effect of MAP is also supported in other fruits such as sweet cherry [44], peach [45], and plum [46]. In our study, MAP treatment significantly reduced the loss of firmness, particularly in the fully and mid-ripe groups. In the groups not treated with MAP, the firmness decreased more rapidly due to increased respiration rate and subsequent enzymatic cell wall destruction. Latifah et al. [47] also reported that slowing respiration delayed fruit softening, which is consistent with our findings. The effect of MAP is based on the suppression of enzyme activities responsible for softening, such as pectinase and polygalacturonase, by creating an environment with high CO₂ and low O₂ [48]. However, since long-term low oxygen conditions can cause anaerobic respiration and off-flavors, the optimizing gas compositions is important [49, 50].

### Soluble solids content, titratable acidity and vitamin C

**Fig. 4:**
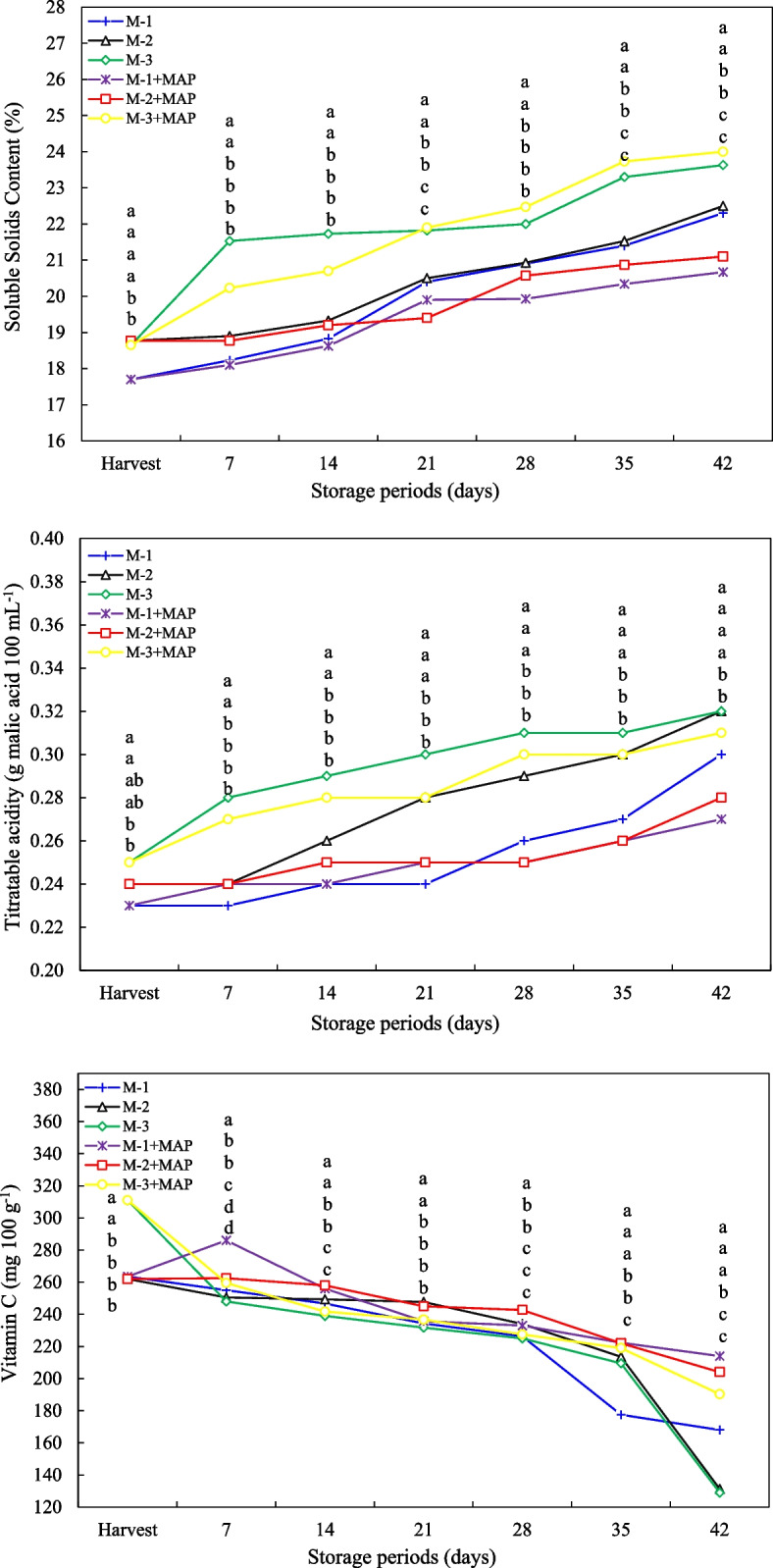
Effect of maturity stage and modified atmosphere packing on SSC, titratable acidity and vitamin C of jujube fruit during cold storage. Means with the same lowercase letter in the same column are no different from each other (Tukey test, *p* ≤ 0.05)

In this study, the changes in SSC, TA, and vitamin C contents of jujube fruits harvested at different maturity levels and treated with MAP were investigated during cold storage(Fig. 4). According to the SSC results, fully maturity fruits (M-3) initially showed higher sugar content than the other groups. (%18.65). The increase in SSC was observed during storage and reached the highest level of 23.63% in M-3 at the end of day 42. MAP treatment, especially in the M-3 + MAP treatment, provided a higher and controlled increase in sugar content (24.00%) while in other groups the SSC increase was limited compared to open storage (p ≤ 0.05) (Fig. 4). These findings demonstrate that MAP supports controlled ripening without disrupting the fruit's metabolic processes. In terms of TA, the highest initial acidity (0.25 g 100 mL^−1^) was detected in fully ripe fruits (M-3). TA increased in all treatments throughout storage, reaching 0.32 g 100 mL^−1^ by day 42, particularly in M-2 and M-3. MAP treatment limited the increase in TA, particularly in groups M-1 and M-2, and contributed to the preservation of organic acids. However, this effect was limited in the M-3 + MAP treatment (p ≤ 0.05) (Fig. 4). Regarding vitamin C content, initially fully ripe fruits (M-3) showed the highest value (311 mg 100 g^−1^). Vitamin C levels decreased in all groups during storage, but MAP treatment significantly slowed this loss. On the 42nd day of storage, vitamin C content was higher in MAP-treated fruits than in control groups (p ≤ 0.05) (Fig. 4). The results suggest that the degree of maturity and storage conditions of jujube fruit are determinants of SSC, TA, and vitamin C levels. The increase in sugar content and the partial rise in acidity as ripening progresses are consistent with transformations in the fruit's chemical components [45]. The increase in SSC reflects the conversion of starch and other polysaccharides to sugars, increasing sweetness and consumer acceptance. The fact that MAP controls the SSC increase is associated with slowing respiration and regulating metabolic activities [51]. The observed increase in TA during storage can be explained by the conversion of starch and other carbohydrates to organic acids. The effect of MAP treatment on limiting acidity increases is consistent with slowing respiration and reducing acid breakdown [52]. However, in ripe fruits (M-3), the effect of MAP was limited due to the more stable metabolic processes. It is also thought that condensation due to water loss may affect the increase in acidity. The acceleration of the decrease in vitamin C content with storage time is associated with the fruit being under oxidative stress and continuing enzymatic degradation [53]. MAP treatment also reduces oxidative degradation and respiration rate by creating a low-oxygen, high-carbon dioxide environment, thus slowing ascorbic acid loss [54]. This protective effect has been similarly reported in other fruits such as pomegranate, strawberry, and sweet cherry. The better preservation of vitamin C content, especially in semi-ripe fruits (M-2 + MAP), is consistent with the effects of maturity level on antioxidant capacity and tissue integrity [55].

### Bioactive compounds

**Fig. 5:**
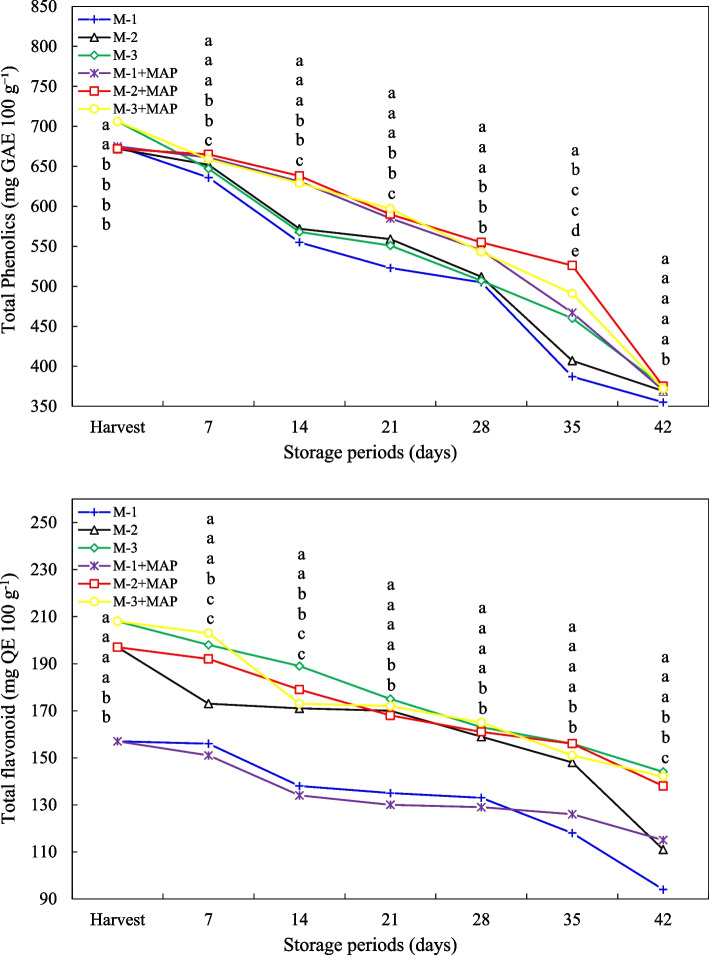
Effect of maturity stage and modified atmosphere packing on total phenolics and total flavonoids compounds of jujube fruit during cold storage. Means with the same lowercase letter in the same column are no different from each other (Tukey test, *p* ≤ 0.05)

In this study, the changes in total phenolic compounds, total flavonoids, and antioxidant activities were investigated in MAP-treated fruits harvested at different maturity stages during storage. In terms of total phenolic compounds, fully maturity fruits (M-3) at harvest had the highest phenolic content (706 mg GAE 100 g^−1^ fw). In the less maturity fruits M-1 and M-2, phenolic contents were measured as 675 and 672 mg GAE 100 g^−1^ fw, respectively. The decrease in phenolic compounds was observed in all treatments throughout the storage period, but at the end of day 42, group M-3 (375 mg GAE 100 g^−1^ fw) still retained the highest phenolic content. MAP treatment delayed the degradation of phenolic compounds and maintained higher phenolic contents in all maturity groups, especially during early storage (p ≤ 0.05) (Fig. 5). The findings revealed that total phenolic compounds increased as maturity progressed in jujube fruit. This is consistent with the accumulation of phenolic compounds during fruit ripening and the activation of biosynthetic pathways [56]. However, phenolic compounds naturally decrease during cold storage. This decrease can be explained by the degradation of phenolic compounds by oxidative enzymes, their polymerization, and oxidative degradation through enzymatic reactions. MAP treatment slows the oxidative degradation of phenolic compounds and preserves the biochemical structure of the fruit [49]. The low-oxygen, high-carbon dioxide environment contributes to the preservation of phenolic content by limiting the oxidative degradation and enzyme activity of phenolic compounds. This finding suggests that MAP treatment allows for better preservation of phenolic compounds during storage. Especially in mid maturity fruits (M-2 + MAP), the preservation of high phenolic content for a longer time indicates that the biochemical stability of the fruit is more suitable at this maturity stage [57].

**Fig. 6:**
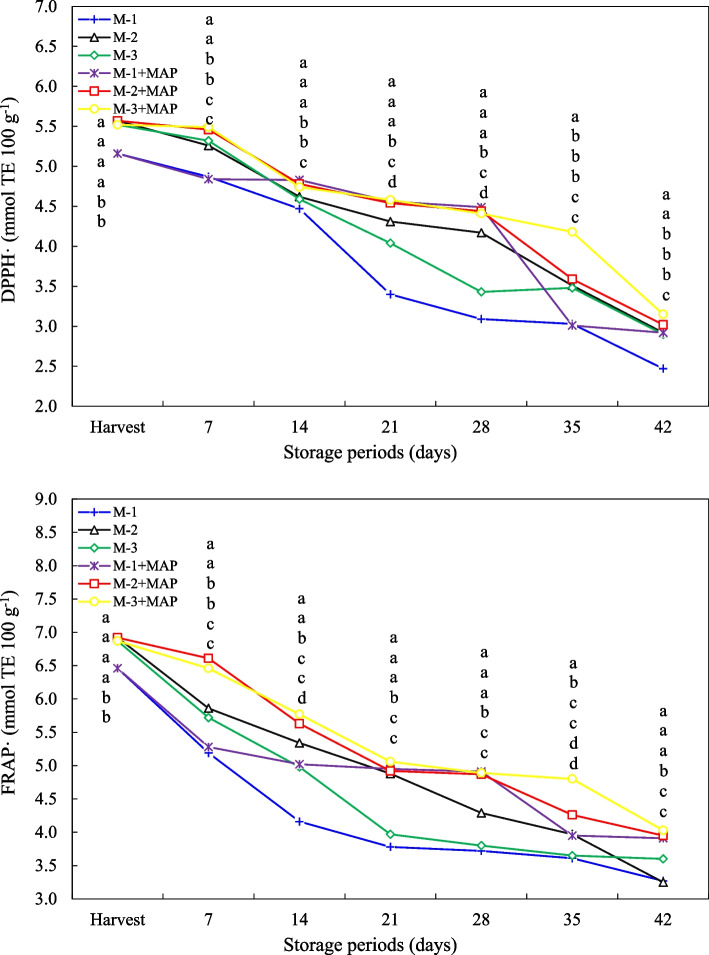
Effect of maturity stage and modified atmosphere packing on antioxidant activities (DPPH and FRAP assays) of jujube fruit during cold storage. Means with the same lowercase letter in the same column are no different from each other (Tukey test, *p* ≤ 0.05)

According to DPPH and FRAP test results, the antioxidant activity tended to decrease over time in all treatments. This decrease was particularly pronounced in FRAP values and occurred more rapidly in the fruits not treated with MAP. For example, in the M-1 group, the FRAP value was 6.46 mmol TE 100 g^−1^ fw at harvest, but this value decreased to 3.27 by day 42. Similarly, in the DPPH test, antioxidant activity decreased in all groups with increasing storage time. However, this decrease was slower in the MAP-treated groups. Considering the effect of maturity stage, the highest antioxidant activity at harvest was detected in groups M-2 (6.92 mmol TE 100 g^−1^ fw) and M-3 (6.87 mmol TE 100 g^−1^ fw) according to both DPPH and FRAP tests. However, as maturity level increased, the antioxidant activity decreased more rapidly during storage. The values for all maturity groups converged, particularly in fruits not treated with MAP after day 35. This demonstrated that MAP provided the significant advantage in preserving antioxidant activity. According to FRAP values, the highest value of 4.03 mmol TE 100 g^−1^ was maintained in the M-3 + MAP group at day 42, significantly higher than that of the M-3 group without MAP (3.60 mmol TE 100 g^−1^ fw) at the same maturity. In the DPPH test, the highest antioxidant activities were measured on days 7, 14, 21, and 28, especially in the M-2 + MAP and M-3 + MAP groups. These findings are consistent with the results obtained in the FRAP test; reducing power increased with maturity and was better preserved during storage with MAP treatment (p ≤ 0.05) (Fig. 6). These findings are consistent with previous studies reporting that MAP application slows the loss of antioxidant activity in cornelian cherry fruit harvested at different maturity stages [58]. This protective effect of MAP treatment is explained by the slowing of respiration rate in the fruit [59].

The similar trends were observed in terms of flavonoids content. The M-3 maurity stage had the highest total flavonoids content value, but there was the decrease in flavonoids content in all treatments during the cold storage period, but MAP treatment significantly limited these losses (p ≤ 0.05) (Fig. 5). This supports the effect of MAP in preserving the biochemical structure of the fruit [58]. Our study showed that phenolic compounds, flavonoids and antioxidant capacity increased as maturity progressed and MAP treatment was effective in preserving these values. These results are consistent with the findings of researchers [56]. In particular, it is known that MAP treatment reduces the oxidative destruction of phenolic compounds by decreasing the oxygen level and increasing the carbon dioxide level [49]. Considering to MAP treatment, it was observed that the harvesting and storing jujube fruit, especially at the M-2 maturity stage, provided advantages in preserving antioxidant capacity for a long time due to both the initial high phenolic content and good storage performance. This finding similarly emphasizes the importance of determining the optimal maturity stage and storage conditions in other fruits [36].

## Conclussion

This study comprehensively revealed the interaction of different maturity stages and MAP treatment on quality traits, physiological changes and preservation of bioactive compounds during cold storage in jujube (*Ziziphus jujuba* Mill.) fruit. The findings show that both fruit maturity level and MAP treatment have decisive effects on quality parameters. MAP treatment significantly slowed down quality losses such as weight loss, fruit softening, decrease in vitamin C and total phenolic compounds at all maturity levels. The balancing effect of MAP treatment on metabolic activities was determined in respiration rate and gas exchange parameters. Especially in more advanced ripeness stages such as M-2 and M-3, MAP was more successful in preserving antioxidant activity (DPPH, FRAP) and total flavonoids content. It was also demonstrated that MAP preserved the quality sustainably in terms of color, firmness and flavor components. As a result, MAP treatment in cold storage of jujube fruit stands out as an effective method in terms of reducing quality losses and preserving functional compounds. However, in order to obtain the most appropriate quality criteria, it is recommended that the fruit should not be harvested at a very early or very late maturity stage, but ideally collected at the mid-maturity level (M-2) and treated with MAP. This approach both extends the storage life and contributes to consumer satisfaction by preserving the superior quality properties in terms of marketability. The study findings provide important data for the sustainability of the jujube fruit supply chain and increasing its commercial value, and also contribute to fill the gaps in the literature on postharvest technologies.

## Data Availability

All data generated or analyzed during this study are included in this published article.

## Abbreviation

DPPH: 2,2-Diphenyl-1-picrylhydrazyl
FRAP: Ferric Reducing Antioxidant Power
FW: Fresh weight
GAE: Gallic acid equivalent
MAP: Modified atmosphere packaging
PG: Polygalacturonase
PME: Pectin methyl esterase
PPO: Polyphenol oxidase
SSC: Soluble solids content
QE: Quercetin equivalent
XET: Xyloglucan endotransglucosylase
